# Green Extraction of Antioxidants from Different Varieties of Red Grape Pomace

**DOI:** 10.3390/molecules20069686

**Published:** 2015-05-26

**Authors:** María José Otero-Pareja, Lourdes Casas, María Teresa Fernández-Ponce, Casimiro Mantell, Enrique J. Martínez de la Ossa

**Affiliations:** Department of Chemical Engineering and Food Technology, Faculty of Science, University of Cadiz, International Agri-food Campus of Excellence, ceiA3, Box 40, 11510 Puerto Real, Cadiz, Spain; E-Mails: mariajose.otero@uca.es (M.J.O.-P.); teresafernandez.ponce@uca.es (M.T.F.-P.); casimiro.mantell@uca.es (C.M.); enrique.martinezdelaossa@uca.es (E.J.M.O.)

**Keywords:** high pressure extraction, pressurized liquid extraction, grape pomace, anthocyanins, antioxidant activity, phenolic compounds

## Abstract

The extraction yield, phenolic content, anthocyanin content and antioxidant activity of extracts from different varieties of red grapes, Cabernet Sauvignon, Merlot, Petit Verdot, Syrah, Tempranillo and Tintilla, using pressurized green solvents have been analyzed. Two techniques were studied and compared: supercritical fluid extraction (SFE) with CO_2_ + 20% ethanol and pressurized liquid extraction (PLE) with either ethanol, water or an ethanol/water mixture as the extraction solvents. The Petit Verdot variety allowed the highest global and phenolic yield, and antioxidant activity. The best conditios for PLE obtained from the experimental design and kinetic study were 50% ethanol/water as the pressurized solvent at 90 bar, 120 °C, a flow rate of 5 g/min and, an extraction time of 90 min. A statistical analysis of variance has been performed and it was found that temperature is the only variable that has a statistical influence on the extraction yield. The antioxidant activity levels of the extracts are very promising and they are similar to those obtained with the antioxidant tocopherol.

## 1. Introduction

Wine production is of great importance in agro economic activities. The world grape production in 2012 exceeded 69 million tons and Europe was the largest producer of wine, with 66% of the total world production [1]. The solid wastes generated by the wine industry represents between 25%–30% of the material used and it consists mainly of grape pomace (containing seeds, pulp, stem and skin) [2,3]. It is well known that high quantities of valuable compounds like dietary fiber, oils from the seeds, anthocyanins and phenolics compounds still remain within the grape pomace after processing [3,4]. The phenolics, such as resveratrol, have great potential due to their antioxidant capacity and health benefits against coronary diseases by the inhibition of LDL (low-density lipoproteins) and other chronic diseases, like cancer, diabetes and neurodegenerative disorders [3,4].

In addition, from the economic point of view, the market of these compounds have been increased in the recent years by the increasing consumer demand for the use of more natural antioxidant compounds, achieving the value of US$30 billions, based on 2008 grape wine production data [3]. In this sense, the valorization and reuse of these wastes from the wine-making industry would have a significant environmental and economic impact, and this possibility has been studied by several authors [3,4,5,6,7].

In recent years, numerous methodologies for the extraction of compounds of relatively high polarity have been developed in an effort to displace conventional solvent extraction techniques. These novel alternative techniques significantly reduce solvent consumption and increase the speed of the extraction by simplifying the process.

Supercritical fluid extraction (SFE) is an efficient technique that is widely applied in the separation of active compounds from herbs and other plants [8]. This technique is appreciated due to the very high solvent power and the distinctive physicochemical properties of supercritical fluids (SCFs). The relatively low viscosity (near to the gas) and the high diffusivity of SCFs help to penetrate the porous solid materials more efficiently than liquid solvents, thus resulting in faster and more efficient extractions. For example, conventional solid-liquid extractions lasting several hours or even days can be achieved in ten minutes on using supercritical fluids [9].

The first scientific work on SFE from grape residues concerned the use of carbon dioxide modified with 5% methanol at 350 bar and 50 °C for the extraction of phenolic compounds [10]. The recovery of resveratrol from grape skin was also optimized by using CO_2_ + ethanol as the solvent system [11]. Chafer *et al.* evaluated the SFE of polyphenols from five grape skin varieties and reported that the most suitable conditions were 60 °C, 250 bar and 20% ethanol as a CO_2_ modifier [6].

It has been shown in different studies that SFE is selective for phenolics, such as gallic acid, catechin, epicatechin and quercetin and have showed high recovery of this polyphenols from grape pomace [6,12,13]. However, this technique cannot be used to extract high molecular weight polyphenols, such as proanthocyanidins, which were more easily extracted by conventional extraction [12]. 

PLE is based on the use of conventional liquid solvents at subcritical conditions with controlled temperature and pressure. With respect to conventional extraction techniques, PLE has the advantage of using less solvent and the extraction is carried out in a shorter time. PLE is widely used for the extraction of antioxidant compounds from winery residues and other natural products [14,15,16,17]. Piñeiro *et al.* compared catechin and epicatechin extraction from tea leaves and grape seeds using ultrasound-assisted extraction (UAE) and PLE [15]. In addition, different solvents, such as water, ethanol and methanol, have been evaluated as hot pressurized solvents for the extraction of anthocyanins and phenolic compounds from grape skin [16,17,18].

Several other methods have been applied for extraction of antioxidants from plant matrices, one such novel process being microwave-assisted extraction (MAE). The advantage of PLE and SFE over MAE is the applicability at different scales. PLE and SFE can be applied to systems on various scales, from the laboratory scale (a few grams) to the pilot plant scale (several hundred grams of samples), through to the industrial scale (tons of raw material) [19,20]. In addition, for winery residues, comparative studies have shown that PLE was more efficient than conventional solvent extraction, MAE and UAE for the recovery of high levels of phenolics from grape pomace and grape skin [5,16]. On the other hand, several studies have demonstrated the economic viability of SFE and PLE for the extraction of winery residues [19] and other raw materials [20].

A great variety of phenolic compounds have been extracted under superheated pressurized conditions from white and red grape skins. Phenolic acids (caffeic acid, gallic acid and protocatechuic acid) and flavonols (catechin, epicatechin and gallate derivatives) were detected, but pyroanthocyanin was also tentatively identified [5]. However, there has been a marked increase in the number of crops for the production of red wines in southern Europe. Many different varieties are grown and these include Tempranillo, Syrah, Cabernet Sauvignon and others. The by-products have already been used for the production of antioxidants using high pressure techniques. Campos *et al.* [21], Tünde *et al.* [22], and previous studies by Mantell *et al.* [23] and Casas *et al.* [24] are some examples. 

As have been aforementioned, several studies have showed that both techniques, SFE [6,12,13] and PLE [14,15,16,17,18], are successfully used to recovery phenolic compounds from grape pomaces, but there are no comparative studies of these techniques to evaluated the efficiency to extract anthocyanins and phenolics from this raw material. Therefore, the aim of the work described here was to evaluate the effect of different experimental parameters, such as pressure, temperature and extraction solvent on the SFE and PLE from different varieties of red grape pomace (Petit Verdot, Tintilla, Syrah, Cabernet Sauvignon, Merlot and Tempranillo). The extracts were analyzed according to the global extraction yield, total phenolic content, total anthocyanin content and antioxidant activity. In addition, the effects of the extraction time and flow rate for PLE were evaluated at the best extraction conditions.

## 2. Results and Discussion

### 2.1. Variety Selection

In order to analyze the influence of red grape varieties for the preparation of a product with a high antioxidant capacity, a series of experiments were designed in which the raw material was varied. Two extraction methods, namely Supercritical Fluid Extraction (SFE) with carbon dioxide with 20% of ethanol and PLE with ethanol, were tested and both the extraction yield and antioxidant capacity were analyzed. For SFE, several authors have reported that 20% of co-solvents is efficient enough to the high extraction yields of anthocyanins and polyphenols from red grape pomace and other raw materials [6,23]. Raising the percent of ethanol as modifier to CO_2_ up to 15%–20% have not showed a significant increase in the yield or the phenolic extraction from grape skins [6,21]. 

**Figure 1:**
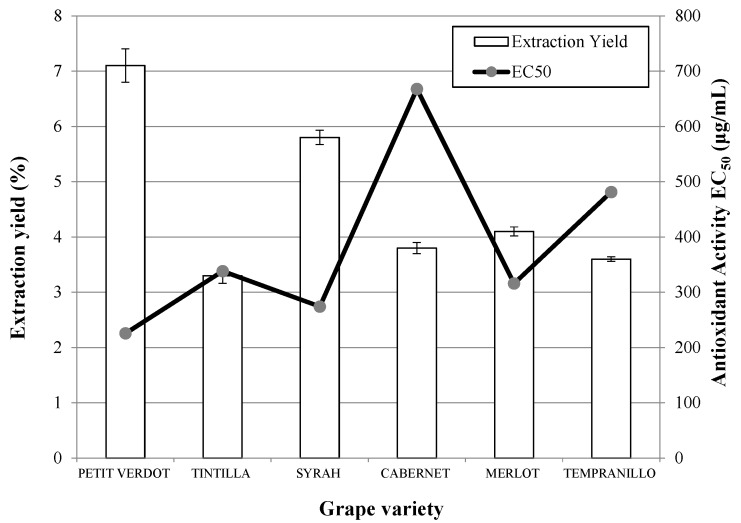
Extraction yield and antioxidant activity obtained by supercritical fluid extraction (SFE) with CO_2_ + 20% ethanol as co-solvent (100 bar, 55 °C, 20 g/min CO_2_, 5 g/min ethanol, 3 h) from different grape pomace varieties.

The results obtained in the SFE at 100 bar, 55 °C and flow rate of 25 g/min are shown in Figure 1 and Table 1.

**Table 1:** Extraction yields of anthocyanins and phenolic compounds expressed as mg/g dry grape pomace.

Variety	SFE CO_2_ + 20% EtOH ^a^		PLE Ethanol ^b^	
	AY ^c^	PY ^d^	AY	PY
Petit Verdot	0.3 ± 0.1	2.5 ± 0.1	16.0 ± 1.0	28.9 ± 1.3
Tintilla	3.8 ± 0.1	4.5 ± 0.1	49.7 ± 2.8	15.5 ± 0.2
Syrah	3.2 ± 0.3	3.6 ± 0.2	38.3 ± 0.6	24.4 ± 0.2
Cabernet	0.1 ± 0.1	2.3 ± 0.3	11.1 ± 1.2	23.8 ± 0.1
Merlot	0.2 ± 0.1	2.1 ± 0.1	10.1 ± 0.1	22.4 ± 0.1
Tempranillo	2.0 ± 0.2	2.2 ± 0.1	30.9 ± 1.0	19.6 ± 0.1

^a^ SFE at 100 bar and 55 °C for 3 h; ^b^ PLE at 120 bar and 100 °C for 3 h; ^c^ Yiel of anthocyanins expressed as mg malvin chloride/g dry grape pomace; ^d^ Yield of phenolics expressed as mg gallic acid equivalent/g dry grape pomace.

It can be seen from this figure that the best extraction yields were obtained with Petit Verdot, followed by Syrah and Merlot varieties. The lowest yields were obtained with Tintilla, Tempranillo and Cabernet Sauvignon, with values of just over half those of the aforementioned varieties. In contrast to the abovementioned, Table 1 shows the SFE from Tintilla and Syrah presented higher recovery of anthocyanins and phenolics than the other grape varieties. The addition of higher concentrations of ethanol as CO_2_ modifier (20%) favored the extraction yield of phenolic compounds in comparison with previous studies using only 4.5% of ethanol (0.2–0.3 mg gallic acid/g dry grape skin) [25].

**Figure 2:**
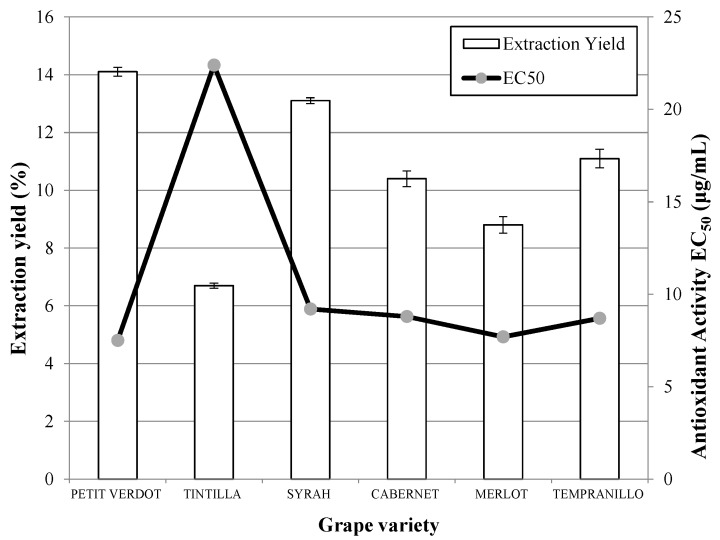
Extraction yield and antioxidant activity obtained by pressurized liquid extraction (PLE) whit ethanol as extraction solvent (120 bar, 100 °C, 10 g/min ethanol, 3 h) from different grape pomace varieties.

The antioxidant activities of the extracts were analyzed and lower EC_50_ values (associated with a higher antioxidant activity) were obtained for the varieties Petit Verdot followed by Syrah (Figure 1). In this case, the antioxidant capacity of the Cabernet Sauvignon samples was the lowest. Comparison of the antioxidant capacities of the extracts obtained in this work with data published previously by other authors shows that the antioxidant capacity of the extracts obtained in this work are lower and are still far from that obtained for the standard (+)-α-tocopherol (EC_50_ = 6.17 ug/mL) [26]. As a consequence, PLE was explored, as it is considered to be an efficient technique for the extraction of polar or slightly polar compounds from different natural materials [16,26,27].The results obtained in PLE experiments using ethanol for the different varieties of grapes analyzed are presented in Figure 2. Once again, the highest global yields were obtained when Petit Verdot was used, followed by Syrah and Tempranillo. In this case, the lowest global yield was obtained when the extraction was carried out on the Tintilla variety. However, according to the anthocyanins and phenolic recovery, Tintilla, Syrah and Tempranillo showed higher yields of anthocyanins whereas Petit Verdot, Syrah and Cabernet presented the higest phenolic yields (Table 1).

As far as the antioxidant capacity is concerned, the highest antioxidant capacities were obtained in the extraction on the Petit Verdot variety. The results obtained with Merlot, Tempranillo, Cabernet Sauvignon and Syrah were very similar and presented values of EC_50_ close to (+)-α-tocopherol between 7.5 and 9.2 μg/mL. Nevertheless, there are significant differences with the Tintilla grape, which gave the lowest results (higher EC_50_).

Comparison of the yields and antioxidant capacities of the extracts obtained using SFE with carbon dioxide and 20% of ethanol as co-solvent and PLE with ethanol shows that the best results were achieved when the extraction was conducted with PLE. In some cases, double the global yield was obtained in comparison to the SFE method and around 10 fold was increased the yield of anthocyanins and phenolic compounds. The yield of anthocyanins and phenolics was in agreement as reported previously for different grape pomaces’ varieties; however, it depends on the origin of the varieties, the genetic variation and the cultivation conditions [7,16]. Similarly, the antioxidant activity results are significantly higher than those shown in Figure 1 and they are comparable to those described by other authors, such asBozan *et al.* [28], who reported EC_50_ values of approximately 3 µg/mL for Merlot and Cabernet samples, and Tounsi *et al.* [29], who reported values of 6.8 µg/mL for Carignan and 30 µg/mL for Syrah seeds, amongst others.

Table 2 shows the concentrarion of anthocyanins and phenolic in the extracts obtained by SFE and PLE from the different varieties of grape pomace. In all the cases, the contents of anthocyanins and polyphenols of PLE extracts were higher than those reported for the extracts obtained by SFE. The PLE extracts obtained from Merlot gave the highest concentration of phenolics (254.6 ± 2.6 mg GAE/g extract) followed by Tintilla, Carbernet and Petit Verdot, which also presented high enough phenolic content. However, Tintilla extracts obtained by PLE presented a significant concentration of anthocyanins that was superior to the others grape varieties.

**Table 2:** Concentration of anthocyanins and phenolics in extracts obtained by SFE and PLE from different varieties of red grape pomace.

Variety	SFE CO_2_ + 20% EtOH ^a^		PLE Ethanol ^b^	
	TAC ^c^	TPC ^d^	TAC	TPC
Petit Verdot	4.9 ± 0.3	34.5 ± 0.4	113.8 ± 7.1	204.9 ± 9.4
Tintilla	116.1 ± 2.0	135.7 ± 0.8	741.9 ± 41.7	231.6 ± 3.5
Syrah	55.1 ± 4.6	62.7 ± 0.7	292.5 ± 4.3	186.3 ± 1.3
Cabernet	3.5 ± 0.1	59.7 ± 1.8	107.0 ± 11.3	228.4 ± 0.9
Merlot	4.5 ± 0.2	52.2 ± 1.1	114.6 ± 0.2	254.6 ± 1.0
Tempranillo	56.8 ± 1.3	59.0 ± 0.9	278.3 ± 9.3	177.1 ± 1.3

^a^ SFE at 100 bar and 55 °C for 3 h; ^b^ PLE at 120 bar and 100 °C for 3 h; ^c^ Total anthocyanins content in extracts expressed as mg malvin chloride/g dry extract; ^d^ Total content of phenolics in extracts expressed as mg gallic acid equivalent/g dry extract.

However, a direct relationship between the total phenolics and the antioxidant activity of the extracts was not observed. Different authors have also reported similar results considering that the antioxidant activity depends on the quality of phenolic compounds. Besides, the antioxidant capacity of the extracts could also be affected by the synergistic effects caused by the interactions of antioxidant compounds and the presence of other non-phenolic compounds [30,31].

Therefore, considering that Petit Verdot grape pomace showed the highest extraction yield and extracts with high antioxidant capacity as well as adequate total phenolic content, this variety and PLE as extraction technique were selected for the subsequent experiments aimed at identifying the best process operating conditions.

### 2.2. Extraction Conditions

#### 2.2.1. Solvent System

On the basis of the results obtained in the previous analysis, the highest extraction yield and the best antioxidant activity were accomplished with the same method (PLE) and with the same grape pomace variety (Petit Verdot).

PLE is increasingly being used as an alternative to carry out environmentally friendly green extractions since it avoids the use of large amounts of solvents, which also provides significant advantages in process automation and sample preparation. In regard to the solvent employed in the extraction, ethanol (EtOH) has been studied as one of the more environmentally friendly solvents (“green” solvent) and it is recognized as safe according to the European Food Safety Authority (EFSA) and FAO/WHO Expert Committee on Food Additives [32,33]. In an effort to reduce the consumption of organic solvents, reports by Štavíková *et al.* [15] and Aliakbarian *et al.* [34] have shown that PLE is favorable for the extraction process on grape skin. Moreover, it has been reported that ethanol/water mixtures are environmentally favorable compared to pure alcohol [35].

**Figure 3:**
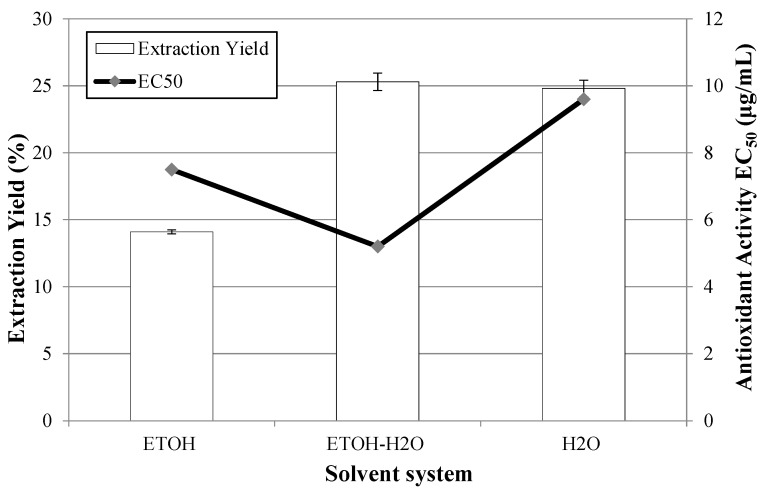
Extraction yields and antioxidant activities, obtained by PLE from Petit Verdot grape pomace using different solvent systems (120 bar, 100 °C, 10 g/min solvent, 3 h).

In order to select the most suitable solvent system in this work, a study was carried out to evaluate PLE procedures with different solvent composition, namely pure ethanol, ethanol/water (50:50) and water, and the results for the global yield and antioxidant activity are represented in Figure 3.

It was found out that the system with the ethanol/water mixture gave the highest extraction yield and the extract with the highest antioxidant activity; for this reason, this solvent was chosen to optimize the extraction method. The performance of this system can be explained because with a dual mixture, particularly a mixture of an organic solvent and water, the extraction efficiency is improved because the organic solvent enhances the solubility of the analyte and water increases the analyte desorption [35].

Furthermore, the analysis of the total phenolic content for the hydroalcoholic mixture (497.32 ± 4.93 mg GAE/mg extract) was superior to that obtained using pure ethanol (204.92 ± 9.43 mg GAE/mg extract) and water (334.61 ± 9.16 mg GAE/mg extract).

Other studies have also shown that for PLE, an increase in the percentage of ethanol has a negative effect on the extraction from grape skin or grape pomace and hydroalcoholic mixtures are more favorable for anthocyanins and polyphenols extraction [23,27,36].

#### 2.2.2. Influence of Extraction Parameters

Temperature and pressure both play a significant role in the extraction process and, as a result, these parameters were studied in order to select the best operating conditions.

**Figure 4:**
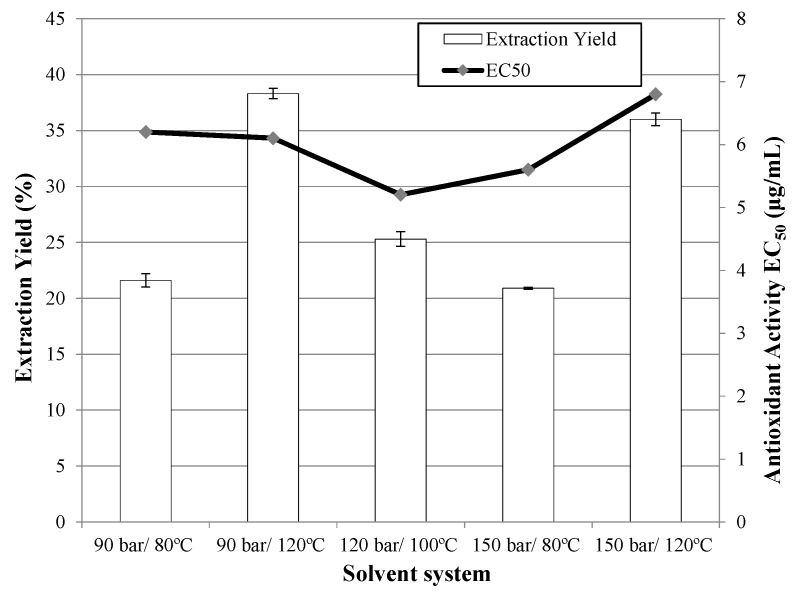
Extraction yield and antioxidant activity obtained by PLE with ethanol/water (50:50) from Petit Verdot grape pomace under different extraction conditions at a flow rate of 10 g/min during 3 h of extraction.

The effects of temperature were studied considering that high temperatures favor extraction efficiency [37]; although this might cause degradation of thermo-labile compounds [38,39]. Three extraction temperatures were tested: 80 °C, 100 °C and 120 °C. It can be observed in Figure 4 that higher temperatures gave higher yields and, therefore, the highest extraction yield was obtained at 120 °C, although the best antioxidant activity was achieved with the extract obtained at 100 °C.

The use of higher pressure led to an improvement in the extraction because the solvent makes contact with the analyte more easily. Three values were analyzed: 90 bar, 120 bar and 150 bar. It can be deduced that the effect of pressure depends on the temperature; hence at a fixed pressure, both extraction yield and antioxidant activity are determined by the temperature. Nevertheless, there are very few differences between the EC_50_ values, and extraction efficiency is, consequently, the decisive result.

It can be seen in Figure 4 that a considerably higher extraction yield is obtained at 120 °C and, at this temperature, the highest antioxidant activity is achieved at 120 bar.

These results are consistent with those of Hawthorne and Miller [40], who first studied PLE with water and showed that temperature has a predominant effect over pressure.

**Figure 5:**
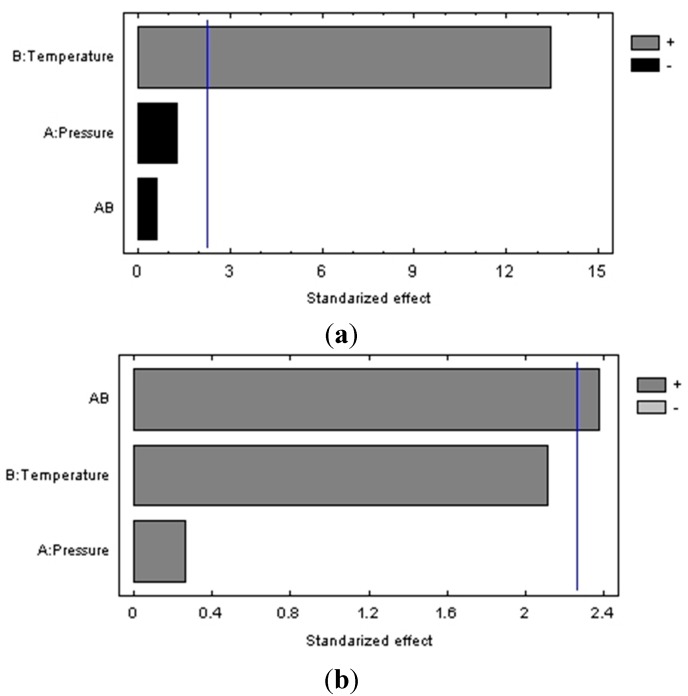
Pareto diagram (**a**) for the global extraction yield (**b**) for the antioxidant activity, obtained by PLE with ethanol/water (50:50) from Petit Verdot grape pomace.

The extraction yields and antioxidant activities obtained for different temperatures and pressures were statistically analyzed. Regression analysis was performed on the experimental data and the coefficients of the model were evaluated for significance. It was observed in the Pareto diagram (Figure 5) that temperature is the only factor that had a significant influence on the extraction yield (*p* ≤ 0.05).

The relationship between temperature and pressure for the global extraction yield is represented by Equation (1).

Y = −16.9 + 0.041667 × P + 0.4775 × T − 0.000666667 × PT
(1)

where Y represents global extraction yield; P the pressure (bar), and T the temperature (°C).

#### 2.2.3. Extraction Kinetics

The flow rate is an important variable in the design of extraction processes. Excessive solvent flow rates lead to insufficient loading of the solvent and, in severe cases, compacting of the bed, which results in preferential pathways through the bed and causes inefficient extraction. On the other hand, low flow rates lead to unprofitable processes due to the long extractor residence times.

**Figure 6:**
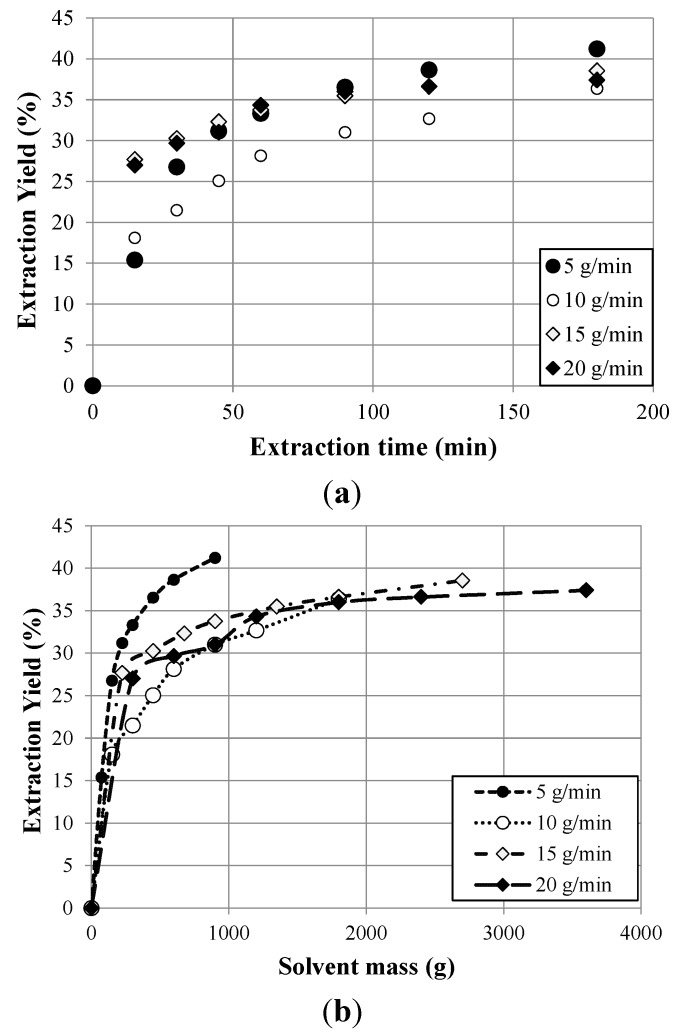
Kinetic extraction in terms of extractions time (**a**) and solvent mass (**b**) for PLE from Petit Verdot grape pomace at 90 bar of pressure and 120 °C of temperature with ethanol/water (50:50) at different flow rates

The global extraction yields obtained with PLE from Petit Verdot variety at 90 bar of pressure and 120 °C of temperature with the mixture ethanol/water (50:50) as solvent at flow rates of 5, 10, 15 and 20 g/min are plotted against extraction time and solvent mass in Figure 6.

It is remarkable that, in the initial stage of the process, the extraction rate is dependent on the flow. At 15 min, a higher extraction yield was obtained in the process with a solvent flow rate of 20 g/min than that obtained at 5 g/min. This behavior is as one would expect; an increase in the flow rate leads to higher yields of extracts. This effect is due to the presence of a large amount of solvent in the operation, a factor that enhances the extraction yield. For extraction times above 50 min, an increase in the flow did not lead to an improvement in the extraction process, despite the fact that solvent consumption was high. In this second part, the slope of the curve decreased and the extraction rate was reduced until a limiting yield was reached, which depended on the total amount of extractable solutes under the fixed operating conditions. As a result, it is advisable to work at a flow rate of 5 g/min for 90 min. Under these conditions a yield of 87% was obtained after 180 min. This behavior is commonly seen using different raw materials [41].

## 3. Experimental Section

### 3.1. Samples and Chemicals

The materials used in this study were the wastes from the vinification of red wine from different varieties. The varieties analyzed were Cabernet Sauvignon, Merlot, Petit Verdot, Syrah, Tempranillo and Tintilla. All of the raw materials were provided by “Bodegas Luis Pérez” (Jerez de la Frontera, Spain). The grape pomaces were obtained immediately after the vinification process and were dried in an oven at 60 °C to constant weight. Prior to the extraction process, the samples were milled in order to reduce the particle size.

Carbon dioxide (99.995%) was supplied by Abello-Linde S.A. (Barcelona, Spain). 2,2-Diphenyl-1-picrylhydrazyl free radical (DPPH). Methanol (HPLC grade), ethanol (HPLC grade) and the other reagents were provided by Panreac (Barcelona, Spain). All experiments were carried out using Milli-Q water (Millipore S.A.S., Molsheim, France). 

### 3.2. Extraction Methods

High pressure extraction tests were carried out in a system supplied by Thar Technology (model SF100, Pittsburgh, PA, USA) provided with an extraction vessel (capacity of 100 mL) and two pumps with a maximum flow rate of 50 g/min. In SFE, two pumps were used (one for carbon dioxide and the other for co-solvent) while in PLE only one pump was employed. Pressure was adjusted at the back pressure regulator and solvent pumps. A thermostatized jacket allowed control of the extraction temperature. The cyclonic separator permitted periodic discharge of the extracted material during the process.

The operating methodology involved loading the extraction vessel with approximately 35 g of the sample, which had previously been homogenized in order to maintain a constant apparent density in all experiments. The extracts were collected in a cyclonic separator and transferred to glass bottles, which were stored at 4 °C with the exclusion of light.

For SFE, the experiments were carried out at a flow rate of 25 g/min for 3 h at 100 bar and 55 °C using CO_2_ + 20% ethanol as solvent. Previous studies have reported that raising the percent of ethanol as modifier to CO_2_ up to 15%–20% have not showed a significant increase in the yield or the phenolic extraction from grape skins [6,21]. 

For PLE, all experiments were carried out at a flow rate of 10 g/min for 3 h. The influence of pressure (P), temperature (T) and ethanol concentration (C) on the extraction process was analyzed. Pressures of 90, 120 and 150 bar, temperatures of 80, 100 and 120 °C, and different concentrations of ethanol in water between 0% and 100% were evaluated. In the case of hydro-alcoholic mixutres, previos studies have showed that 50% ethanol was successful to extract anthocyanins from red grape pomace with similar yields than conventional extraction [35]. The experiments on each sample were carried out in duplicate in order to evaluate the variability of the measurements.

The global extraction yields obtained by SFE and PLE were calculated as the ratio of dry extract to dry raw material and the results are expressed as g extract/g raw material.

### 3.3. Total Phenolic Content

The determination of total phenolic compounds present in the extracts was carried out using an HPLC system 1100 series supplied by Agilent Technologies (Waldbronn, Germany) comprising a degasser, a quaternary pump, an autosampler and a UV/vis detector. 

The extracts were previously filtered and subsequently 20 µL aliquots were injected and analyzed by HPLC using a Synergi Hydro–RP C18 column (150 mm × 3 mm i.d., 4 μm particle size) (Phenomenex, Torrance, CA, USA) with a C18 ODS guard column (4.0 mm × 2.0 mm i.d.). The mobile phase was acidified water containing 0.1% formic acid (A) and acetonitrile with 0.1% formic acid (B). The gradient profile was as follows: 0 min, 0% B; 0.2 min, 0% B; 0.3 min, 7% B; 14.7 min, 8.5% B; 40 min, 19% B; 45 min, 33% B; 48 min, 50% B; 50 min, 95% B; 57 min, 0% B; and 63 min, 0% B. The flow rate was 1 mL/min and the wavelength of detection was set at 278 nm.

Total phenol content was determined as the sum of the peak area of all the phenolic compounds and expressed as mg of gallic acid equivalent (GAE)/mg dry extract based on a calibration curve with gallic acid. Analyses were carried out in triplicate and SD was estimated. Other authors have also used chromatographic methods as an accurate approach to obtain the total phenolic content [31,42].

### 3.4. Anthocyanins Analysis

The analysis of the anthocyanins present in the extracts was performed in and HPLC system provided by Agilent Technologies (Palo Alto, CA, USA) 1100 Series chromatograph. 5% Formic acid in water (v/v) (A) and methanol (B) were used as solvents in this HPLC method. The HPLC gradient program was executed as follows: 98% A to 40% A in 60 min, 40% A to 98% A in 5 min. The entire HPLC run time was 70 min using a flow rate of 1 mL/min.

The resultant extracts were filtered before HPLC assay using a 0.45 µm PTFE filter (Varian Inc., Palo Alto, CA, USA) and 100 µL of the filtered extract was injected into the column (250 mm × 4.6 mm) C18 Hypersil ODS (5 µm particle size) (Supelco). The total content of anthocyanins was calculated as the sum of the peak area of all the compounds detected at a wavelength of 510 nm. The results were reported based on the calibration curve of malvin chloride and expressed as mg of malvin chloride/mg dry extract. The experiments for each extraction were carried out in triplicate in order to evaluate the variability of the measurements. The method above described has also been used by other authors [43,44].

### 3.5. Antioxidant Assay with DPPH

The method used to measure the antioxidant activity of the extracts obtained from the grape pomace was based on the use of DPPH as a free radical. The technique proposed by Brand-Williams *et al.* [45], and modified by Scherer *et al.* [46], is based on the use of free radical 2,2-diphenyl-1-picrylhydrazyl (DPPH•), which absorbs at 515 nm. Reduction of this radical with an antioxidant leads to the disappearance of the absorption at this wavelength. Thus, the decrease in the absorbance allows an assessment of the ability of the compound to scavenge free radicals. 

The 3.9 mL of 6 × 10^−5^ mol/L methanol DPPH solution were added to 0.1 mL of extract methanolic solutions at different concentrations. The blank sample consisted of 0.1 mL of methanol added to 3.9 mL of DPPH solution.

The absorbance of DPPH was monitored spectrophotometrically at 515 nm at 0 min and every 2 min until a steady state was reached. The DPPH concentration (C_DPPH_) in the reaction medium was calculated from a calibration curve determined by linear regression with the following equation:

(2) $$\text{Abs}=12709\times {\text{C}}_{\text{DPPH}}+0.002$$

The percentage of DPPH remaining was calculated as described in Equation (3).

(3) $$\text{\%DPPH remaining}={\text{C}}_{{\text{DPPH}}_{\text{t}}}/{\text{C}}_{{\text{DPPH}}_{0}}\times 100$$

The EC_50_ (efficient concentration providing 50% inhibition) was calculated graphically using a non-linear calibration curve by plotting the extract concentration *vs.* %DPPH remaining on the steady state. The experiments were carried out in duplicate in order to evaluate the variability of the measurements.

### 3.6. Experimental Design

Initially, a preliminary study was conducted to select the most appropriate technique and the best variety of grape pomace. All 6 grape varieties were tested in the two systems studied: supercritical fluid extraction (SFE) with CO_2_ + 20% ethanol as co-solvent and pressurized liquid extraction (PLE) with ethanol. The use of ethanol as a modifier in the supercritical fluid extraction (SFE) is justified since it increases significantly the solubility of the polyphenols in the supercritical phase and improves their extraction. Moreover, ethanol is the most suitable polar modifier due to its volatility and non-toxicity, in addition to its regular use in the pharmaceutical, medical, cosmetic and food industries [47]. The SFE process operating conditions were: 100 bar, 55 °C, flow rate of 25 g/min and 20% (v/v) of co-solvent. In PLE the extraction tests were performed at 120 bar, 100 °C, flow rate of 10 g/min. In each case, the response variables analyzed were the extraction yield (expressed as g extract/100 g of dry matter) and antioxidant activity of the extracts (AA) (expressed as µg/mL of extract).

After selecting the most suitable variety of grape pomace, the PLE process was carried out with different solvents, namely ethanol, water and an ethanol/water mixture (50:50), with the operating conditions listed above kept constant for this technique.

In addition, with the variety of grape pomace and the solvent chosen for the PLE, an experimental factorial design 2^2^ was performed to study the extraction process and to identify the best operating conditions (according to the objective of the present article). The variables selected for the experimental design were pressure (P), with values of 90 and 150 bar, and temperature (T), with values of 80 and 120 °C. Finally, flow rates of 5, 10, 15 and 20 g/min were tested for PLE under the best operating conditions. All experiments were carried out with an extraction time of 3 h.

The results were analyzed using the Statgraphics Plus 5.1^®^ (1994–2001, Statistical Graphics Corp., Princeton, NJ, USA) program to evaluate the influence of the factors on the extraction process and to determine significant differences in the samples for each variable. Empirical correlations were developed in order to predict the influence of extraction conditions on both the extraction yield and the antioxidant activity. Significance levels of factors were defined using *p* = 0.05, so factors and their combinations with a *p*-value < 0.05 have a significant influence on the extraction process with a confidence level of 0.95. The sign associated with each factor indicates positive or negative effect caused by the variable.

## 4. Conclusions

Two high pressure extraction techniques, SFE and PLE, were evaluated from wine industry wastes. The comparative study using different variaties of grape pomace showed PLE using hydro-alcoholic mixture as solvent was more efficient than SFE using CO_2_ + 20% ethanol in terms of both phenolic ontent and antioxidant activity. The global yield and the yield of anthocyanins and phenolic compounds was higher for PLE than SFE. 

The comparison of six different grape varieties showed that Petit Verdot grape pomace provided the highest global and phenolic extraction yield, as weel as strong antioxidant activity using pressurized ethanol as extraction solvent. However, in terms of anthocyanins Tintilla showed the highest yield (49.7 mg/g dry pomace) and total content in the extracts (741.9 mg/g extract).

In addion the experimental design and the kinetic study showed that the highest extraction yield for PLE was obtained at 120 °C and 90 bar using hydro-alcoholic mixtures at a flow rate of 5 g/min for 90 min. Furthermore, from the statistical analysis of variance it was found that temperature was the only experimental parameter that has a statistical influence on the extraction yield from grape pomace by PLE. It was demosntrated that this technique is succesfull to extract antioxidant phenolic compounds from grape pomace in order to increase the value of this winery by-product with potential applications in differents industrial sectors like cosmetics or nutraceutics.
